# Genome-wide localization of mobile elements: experimental, statistical and biological considerations

**DOI:** 10.1186/1471-2164-6-81

**Published:** 2005-06-01

**Authors:** Betsy M Martinez-Vaz, Yang Xie, Wei Pan, Arkady B Khodursky

**Affiliations:** 1 Department of Biochemistry, Molecular Biology and Biophysics and Biotechnology Institute, University of Minnesota, Saint Paul, MN 55108, USA; 2 Biostatistics Department, School of Public Health, University of Minnesota, Minneapolis, MN 55434, USA

## Abstract

**Background:**

The distribution and location of insertion elements in a genome is an excellent tool to track the evolution of bacterial strains and a useful molecular marker to distinguish between closely related bacterial isolates. The information about the genomic locations of *IS *elements is available in public sequence databases. However, the locations of mobile elements may vary from strain to strain and within the population of an individual strain. Tools that allow *de novo *localization of *IS *elements and are independent of existing sequence information are essential to map insertion elements and advance our knowledge of the role that such elements play in gene regulation and genome plasticity in bacteria.

**Results:**

In this study, we present an efficient and reliable method for linear mapping of mobile elements using whole-genome DNA microarrays. In addition, we describe an algorithm for analysis of microarray data that can be applied to find DNA sequences physically juxtaposed with a target sequence of interest. This approach was used to map the locations of the *IS5 *elements in the genome of *Escherichia coli *K12. All *IS5 *elements present in the *E. coli *genome known from GenBank sequence data were identified. Furthermore, previously unknown insertion sites were predicted with high sensitivity and specificity. Two variants of *E. coli *K-12 MG1655 within a population of this strain were predicted by our analysis. The only significant difference between these two isolates was the presence of an *IS5 *element upstream of the main flagella regulator, *flhDC*. Additional experiments confirmed this prediction and showed that these isolates were phenotypically distinct. The effect of *IS5 *on the transcriptional activity of motility and chemotaxis genes in the genome of *E. coli *strain MG1655 was examined. Comparative analysis of expression profiles revealed that the presence of *IS5 *results in a mild enhancement of transcription of the flagellar genes that translates into a slight increase in motility.

**Conclusion:**

In summary, this work presents a case study of an experimental and analytical application of DNA microarrays to map insertion elements in bacteria and gains an insight into biological processes that might otherwise be overlooked by relying solely on the available genome sequence data.

## Background

Insertion elements, the simplest bacterial transposons, are short DNA sequences (700–2500 bp) carrying only genetic information related to their transposition and its regulation [1]. *IS *elements are capable of transposition into many sites within and between bacterial chromosomes and extra-chromosomal elements. The movement of *IS *elements can cause activation or silencing of adjacent genes [2]; chromosomal rearrangements such as deletions, inversions and insertions are also common consequences of *IS *element activity [3]. Due to diverse genetic effects associated with the activity of insertion elements, developing tools to identify and map the location of these DNA sequences in bacterial genomes is essential to advance our understanding of the role *IS *elements play in gene regulation and genome plasticity.

Mapping insertion elements in microbial genomes is important for several reasons. First, the distribution and location of insertion elements in a genome is a potent tool to track the evolution of a bacterial strain [4-7]. Second, *IS *elements are often used as molecular markers to distinguish between closely related bacterial strains. This approach is helpful in epidemiological studies in which the presence and location of a particular insertion element have been used as a marker to track the epidemiology of microbial pathogens [8,9].

Although the information about the genomic locations of *IS *elements is available in public sequence databases, by definition, the locations of mobile elements may vary from strain to strain and within the population of an individual strain [3], and [10]. Thus we need a tool that would not be solely dependent on the existing information about the location of insertion elements, but instead would allow *de novo *mapping of the sequences.

A variety of molecular techniques have been used to map insertion elements in bacteria. These include Southern hybridizations, inverse PCR, and vectorette PCR [11,12]; and [13]. Inverse PCR and Southern hybridizations are very laborious techniques that require further sample processing to determine the location of the insertion sequences. Recently, vectorette PCR has been described as rapid and efficient method to map *IS *elements in the *E. coli *genome [13]. DNA microarrays provide a powerful alternative to the gel-based techniques and allow reliable determination of relative abundances of individual RNA or DNA species in complex mixtures. Most microarray applications attempt to assess the relative abundance of individual nucleic acids species by labeling it (along with others in the mixture) directly, in sequence-independent manner [14-17] and [18]. However, the identification of neighboring sequences using microarrays relies on a sequence-dependent labeling by primer extension from a known sequence [19] and [9]. Raychaudhuri [19] developed and later applied [9] an algorithm using non-parametric discriminant analysis to predict the location of *IS6110 *element within the *Mycobacterium tuberculosis *genome from microarray data. Their algorithm requires two sets of feature training examples: insertion sites and non-insertion sites and the authors used known insertions sites across multiple experiments to generate the examples. However in most real life cases, we do not have multiple experiments and do not know the sites of insertions or non-insertion sites in advance. In this paper, we propose a simple but reliable algorithm to predict the genomic location of insertion DNA sequences based on microarray data without using the prior knowledge about the location of mobile elements.

Most applications of spotted cDNA microarrays rely on two-color competitive DNA hybridization to assess the relative abundance of nucleic acid species represented on the array [14]. Often, however, it is quite difficult to choose an adequate biologically meaningful reference for the two-color hybridization. In such cases especially, the reference serves only to compound the error in the ratio calculation. In this article we investigate the applicability of single channel hybridization for making biologically relevant inferences from microarray data.

We applied the tools presented herein to map the locations of the *IS5 *elements in the genome of *Escherichia coli *K12. We observed heterogeneity in localization of the elements within a population of the strain. The biological implications of this finding are discussed.

## Results

### Genome-wide mapping of *IS5 *elements

Individual clones of the *E. coli *MG1655 were obtained by plating an ATCC culture of the strain whose complete genome sequence was published in 1997 [20]. Liquid cultures of single colonies were used as frozen stocks for subsequent experiments. We carried out mapping experiments with two sets of biological replicates, where each set is represented by several independently grown colonies from two separately frozen stocks of bacteria. We designated these stocks 'A' and 'B', and the sets of biological replicates were A1, A2, A3, A4, A5 and B1, B2, B3, B4, B5, B6. We also carried out a set of control experiments designated as C1, C2 and C3, where no neighbor sequences should've been detected because purified transposon DNA was used as a template in the probe preparation. The *IS *mapping data presented in this paper is publicly available in the NCBI GEO database under the following accession numbers: GSE2697.

Table 1 compares the performance of different test statistics and normalizations in three experiments (control, stocks 'A' and 'B') based on the available sequence data. In this table, the neighbours from sequence data were defined as 5 upstream and 5 downstream genes of an *IS5 *element and the *IS5 *itself, there are eleven known copies of *IS5 *in the *E. coli *genome. Combining this information, we get 11 neighbours per *IS5 *element × 11 *IS5 *copies; this equals 121 neighbours based on sequence data (11*11 = 121). From this table (Table 1), we can see that rank-based statistics (rank product and median rank) perform similarly (the overlap between the lists of top genes identified by rank-product and median rank is about 90%), and are better than intensity-based statistics (Mean, SAM and *t *statistics). This statement is based on the observation that using rank statistics allows us to identify more neighbours in the experimental samples (stocks A and stock B) and fewer neighbours in the controls. In contrast, intensity-based statistics identified more gene neighbours for the control samples and fewer neighbours for the experimental samples. Among the intensity-based test statistics, it is hard to tell which one performs better for these data; however, none of them is better than rank-based methods. Using different normalization methods did not affect the results of rank-based analysis but led to different results than using intensity-based statistics. Consistent with our expectation, whereas only the *IS5 *element itself could be detected in the control experiment, *IS5 *elements and their neighbours can be found when genomic DNA from samples 'A' or 'B' was used as a template. The results of microarray experiment with the genomic DNA from stock 'A' were somewhat more consistent with sequence data than those obtained using DNA from stock 'B'. Based on these comparisons, we used median rank as our test statistic.

**Table 1:** Comparisons of different methods used to identify *IS5 *neighbouring genes from microarray experiments. Results obtained when different test statistics (rank product – RP, median rank – MR, Mean, SAM and *t*-statistics) and normalization methods (global and scale normalization) were applied to experimental data from the control, isolate 'A' and isolate 'B'. These results were also compared to existing genome sequence data. Top 50 to 100 genes were predicted as *IS5 *neighbours from microarray experiments. The neighbours from sequence data were defined as 5 genes upstream and 5 genes downstream of the *IS5 *elements and *IS5 *itself, so there were 121 neighbours based on sequence data. The numbers in this table represent number of genes that overlap between the microarray experiment and the sequence data. The higher the number is, the better consistency between the microarray experiment and the sequence data.

Method		Top 50			Top 100		
		
		control	A	B	control	A	B
Rank – based	RP	13	46	31	15	69	43
	MR	13	45	32	15	70	47

Global Normalization	Mean	20	37	20	23	65	29
	SAM	20	38	24	23	65	29
	t	20	38	24	23	65	31

Scale Normalization	Mean	20	44	21	23	65	23
	SAM	20	40	22	23	63	29
	t	20	16	24	23	30	31

**Figure 1:**
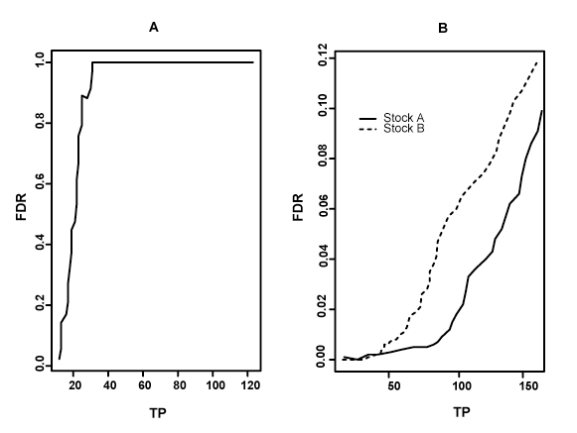
**False discovery rate for median rank statistic**. False discovery rate, FDR, as a function of the number of total (claimed) positive genes, TP. **A**, FDR in the control experiment where fluorescent probe was confined to the *IS5 *sequence. **B**, FDR in the experiment where floarescent probe extended into the *IS5 *neighboring regions.

We used false discovery rate plots to decide the cut-off for claiming the significant genes for median rank statistic. Figure 1 shows that the false discovery rate increases much more rapidly in the control compared to the experiment as the number of claimed positive genes increases. This observation is consistent with our hypothesis that we should find more true positive genes in experiments (including *IS5 *elements and their neighbours) than in controls (where only *IS5 *elements were expected to be detected). For the same number of claimed positive genes, the FDR for stock 'A' was somewhat lower than for 'B': when claiming top 100 genes as significant genes, the FDR for stock 'A' was about 0.02 and for stock 'B' was about 0.06. We regarded these false discovery rates (6 genes per 100) as acceptable and we set the cut-off for selecting significant genes at the top 100 genes.

**Figure 2:**
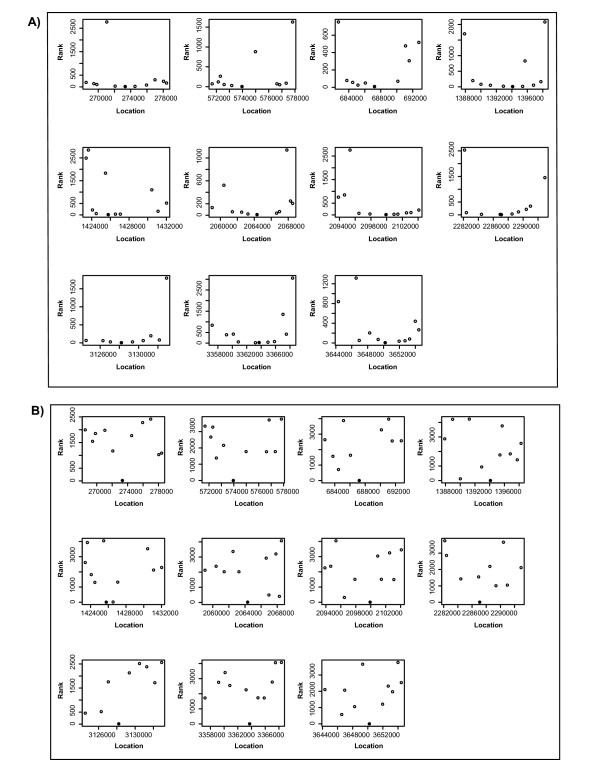
**The ranks of the true neighbours of *IS5 *elements in the *E. coli *genome**. **A**, Experiment. **B**, Control. Rank of fluorescent intensities of target sequences as a function of their positions, relative to the position of an *IS5 *element. All known 11 locations of *IS5 *elements are shown as separate panels. The closed circles represent the *IS5 *elements and open circles represent the neighbours. The lower the rank, the higher probability that the gene is a neighbour of the *IS5 *element. Experiment and control are described in the legend to Figure 1 and in the text.

To determine whether there is any relationship between the proximity of a neighbour to the *IS5 *element and its intensity we constructed "Rank *vs*. Distance" plots for all 11 known *IS5 *positions in the genome (Fig. 2). The open circles represent the neighbouring ORFs and the solid circles correspond to *IS5 *elements. Figure 2A demonstrates that most of the genes in immediate proximity to *IS5 *elements have consistently low ranks, with very few outliers with high ranks. We obtained similar results for both stocks 'A' and 'B' (data not shown). The "Rank *vs*. Distance" plots in Figure 2B clearly demonstrate that there is no association between the rank and the distance in the control. While the ranks of *IS5 *elements are very low, the ranks of expected neighbours are high and random.

100 genes predicted as neighbours of *IS5 *elements by median rank for stock 'A' are listed in Table 2. They include 12 genes that are likely to be false positives as there are no neighbours of those genes in the list of top 100; 4 pairs of possible significant genes with one neighbour present on the list, 12 groups of likely true positive genes with at least 2 neighbours on the top ranking list. Among 12 groups of significant genes (total of 80 genes), 11 groups are expected neighbours of *IS5 *elements based on the genome sequence data. Only one group of genes including *motB*, *motA*, *flhC*, *flhD*, *yecG*, *otsA*, *otsB *are not known neighbours of an *IS5*. It is highly unlikely to find 7 neighbouring genes as significant genes by chance alone. Table 3 lists 100 genes predicted as neighbours of *IS5 *elements by median rank method for stock 'B'. Among these 100 genes, 33 were stand alone genes without a single neighbour on the gene list. We designated them as false significant genes. We identified 12 groups of genes (total of 55 genes) as significant genes, with at least 2 neighbours appearing on the list. Among these 12 groups, 11 were known neighbours of *IS5 *elements. Only one group of three genes (*pspB*, *pspC*, *pspD*) was not previously known to be in the vicinity of an *IS5*. Compared to stock 'A', the number of false significant genes identified as neighbours of *IS *elements in stock 'B' is much higher, which is consistent with the observed differences in false discovery rates (Fig. 1). However, we could identify all known 11 locations of *IS5 *in both stocks. The most pronounced difference between two stocks, in terms of a probability of finding the *IS *element in a new location by chance, was the apparent presence of the transposon sequence proximal to the *flhDC*/*yecG *group of genes.

**Table 2:** The 100 genes predicted as neighbours of *IS5 *elements in the genome of MG1655 isolate 'A'. False significant genes are the genes without any neighbours (5 up- or down-stream neighbours) on this gene list; possible significant genes are the genes with one neighbor on the list; significant genes are the genes with at least 2 neighbours on the gene list. The names in bold are the known *IS5 *elements.

False significant genes	lpxC, yi22_1, b0878, ompF, b1297, flip, ogrK, fruA, b2442, b2639, cpxR, araH
Possible significant genes, strings of neighbours	1. yi22_2, tra8_2
	2. b1578, rspB
	3. yi22_4, yi21_4
	4. yi21_5, yi22_5

Significant genes, strings of neighbours	1. b0255, tra8_1, ykfC, **yi52_1**, ykfD, yagD, insB_2
	2. b0546, b0547, b0548, b0550, b0551, **yi52_2**, b0554, b0555, b0556
	3. gltL, gltK, gltJ, ybeJ, **yi52_3**, b0658
	4. b1328, b1329, b1330, **yi52_4, **b1332, fnr
	5. b1361, b1362, trkG, b1368, **yi52_5, **b1371, b1372, b1374
	6. motB, motA, flhC, flhD, yecG, otsA, otsB
	7. nac, cobT, cobs, cobU, **yi52_6, **b1995, yi22_3
	8. b2028, gnd, **yi52_7, **yefJ, yefI, yefH, yefG
	9. yejM, yejO, b2191, **yi52_8, **narP, ccmH
	10. glcD, glcC, b2981, **yi52_9**, b2983, b2984, b2986
	11. yhcD, yhcE, **yi52_10**, b3219, yhcG
	12. arsB, yhiS, **yi52_11**, slp, yhiF, yhiD

**Table 3:** The 100 genes predicted as neighbours of *IS5 *elements in the genome of MG1655 isolate 'B'. Refer to the legend in Table 2 for details.

False significant genes	araA, b0607, lipA, ybfA, tolR, b0817, ycfJ, b1388, relB, insA_5, flip, b1971, yeeD, b2059, ogrK, ompC, ptsH, b2505, mltB, rpoS, relA, fucK, exuR, yhbY, crp, rhaA, yjdG, tra8_3, yjhG, yjhL, pssR, smf_1, b1965
Possible significant genes strings of neighbours	1. insA_1, yaaC
	2. yi22_1, b0365
	3. b1141, b1146
	4. rspB, rspA
	5. yi22_4, yi21_4
	6. b3254, yhdT

Significant genes, strings of neighbours	1. ykfC, **yi52_1**, ykfD, insB_2, insA_2
	2. b0546, b0547, b0550, b0551, **yi52_2**, b0555
	3. gltK, gltJ, ybeJ, **yi52_3**
	4. pspB, pspC, pspD
	5. b1330, **yi52_4**, b1332, ydaA, fnr
	6. b1368, **yi52_5, **b1371, b1372
	7. cobs, cobU, **yi52_6, **yi22_3
	8. gnd, **yi52_7**, yefJ, yefI
	9. yejO, b2191,**yi52_8, **ccmH
	10. glcC, b2981, **yi52_9, **b2983, pitB
	11. yhcD, yhcE, **yi52_10, **b3219, b3221, sspB, b3238
	12. yhiS, **yi52_11**, yhiF, tnaA

**Figure 3:**
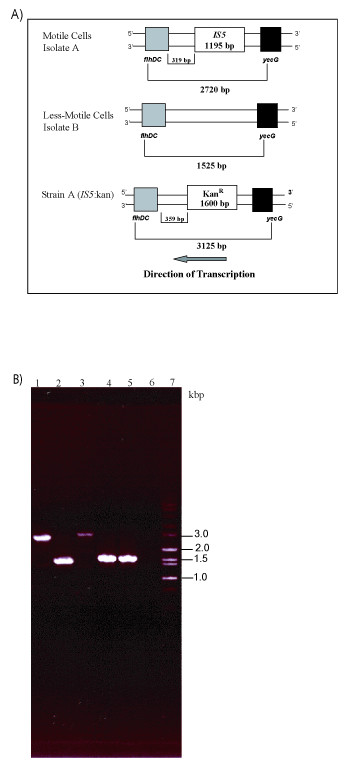
**A**, A diagram of the *flhDC*-*yecG *region in 'A' (motile), isolate 'B' (less motile) and in the *IS5*:kan^r ^mutant. The location of *IS5 *relative to *flhDC *transcriptional start site is shown. The position of the primers used for the verification PCR and the length of the expected PCR products are indicated. The wide arrow at the bottom of the diagram indicates the direction of transcription. **B**, Confirmation of the presence of an *IS5 *element in the *flhDC*-*yecG *region. 1.0% agarose gel shows the products of the PCR reactions confirming the *IS5 *mapping predictions as well as the replacement and deletion of the *IS5 *element by the lambda red recombinase system. The PCR products shown were obtained from reactions done with *flhDC*F3 and *yecG*R primers. Lane1: isolate 'A' (motile); lane2: isolate 'B' (less motile); lane3: *IS5: *kan^r^; lane4: isolate 'AΔ*IS5*'-colony #1; lane5: isolate 'AΔ*IS5*' – colony #2, lane6: no template negative control; lane7: Hi-lo DNA marker ladder. The size of the PCR reaction products is shown in kb.

We confirmed the presence of an *IS5 *element in the *flhDC*/*yecG *intergenic region by PCR. A 2720 bp PCR product was obtained with primers specific for the *flhDC/yecG *intergenic region and chromosomal DNA from stock 'A' as a template. In contrast, when the same primers were used with DNA from stock 'B', only a 1525 bp DNA fragment was obtained (Fig. 3A and 3B). Further DNA sequence analysis confirmed the presence of *IS5 *upstream of *flhDC*, in 'A', while no insertion elements were identified upstream of *flhDC *in 'B'. The inverted repeats of the *IS5 *insertion element were located 319 bp upstream of the reported *flhDC *transcription start site [21] (Fig. 3A). The sequence of the inverted repeats was 100% conserved relative to the reported *IS5 *gene sequence [22]. In addition, a 4-bp target duplication site (5'-TTAG-3') was found flanking the inverted repeats at the *IS5 *insertion sites. These experiments showed that as predicted by our analysis, an *IS5 *element was present in the *flhDC */*yecG *region.

We also examined one of the transposon locations that was likely to be a false significant as it has been suggested by our statistical analysis. PCR analysis of the *fliP *neighbourhood was done with primers specific for the gene regions of *fliO*/ *fliP *and *fliP*/*fliQ*. The size of the PCR products obtained (1000 bp) was the expected size for the gene regions examined based on genome sequence data [20] (data not shown). This indicates that there is no *IS5 *element adjacent to *fliP *and that our assertion that genes present on the list of top ranking candidates without neighbours are likely to be false calls is correct.

Thus the predicted neighbours of an *IS5 *element were consistent with the verification experiments results and the sequence data. If we assume 11 *IS5 *insertion sites based on sequence data and the *flhDC*/*yecG *site based on verification experiments are the only insertion sites in the chromosome of 'A', then there are 11(neighbours)*12 (sites) = 132 neighbours. Assuming all other genes are non-neighbours, and we designated 80 significant genes as predicted neighbours, then the sensitivity of our algorithm is 80/132 = 60.6%. Specificity is 1 and false discovery rate is 0, since all predicted genes are true neighbours.

### Biological implications of the presence of an *IS5 *element upstream of *flhDC*

**Figure 4:**
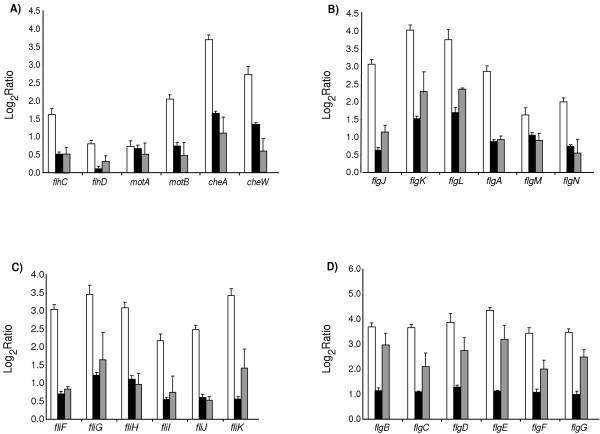
**Relative abundance of flagella transcripts **in isolate 'A' versus 'B' (open bars), in 'A' versus 'AΔ*IS5*' (solid bars), and in 'AΔ*IS5*' versus isolate 'B' (gray bars). The log_2 _(Ratio) values of transcript abundances obtained following lowess smoothing and ANOVA normalization in direct comparisons on whole-genome DNA microarrays are plotted along the Y-axis. The names of the genes are shown under the horizontal axis. Error bars indicate the standard error for at least six experimental replicas.

So far we have demonstrated that our method allowed determining genome-wide distribution of insertion elements and that our analysis is sensitive enough for the purposes of differential localization of transposons in genomes of *E. coli *isolates. Differential localization of insertion elements maybe reflective of different history of isolates or of heterogeneity in a cell population. We confirmed that isolates 'A' and 'B' are indistinguishable by genetic fingerpriniting [23] and by comparative genomic hybridization on arrays [15] (data not shown) and that original population of an ATCC strain was split almost one-to-one between an 'A' type cells containing an *IS5 *element in the vicinity of *flhDC*/*yecG *and a 'B' type without an element. Next, we examined whether two types of cells that we distinguished by a location of an *IS5 *marker may have phenotypic differences associated with an insertion element. We directly compared expression profiles of two isolates using whole-genome DNA microarrays. Following the lowess smoothing and ANOVA normalization [24], we identified 369 genes at a false discovery rate of less than one gene per list [25], whose transcripts appeared to be differentially abundant between the two isolates <the gene expression data presented in this paper is publicly available in the NCBI GEO database under the following accession numbers: GSE2694>. One functional group dominated the list of significant genes: 54 genes classified as being involved in motility and chemotaxis "consumed" 90% of the variance of the entire list of significant genes. On average the flagella genes in isolate 'A' were 9-fold more abundant than in isolate 'B' (Fig. 4). The levels of expression of *flhD *and *flhC*, genes encoding a master-regulator of the motility and chemotaxis regulon and situated in immediate proximity to an *IS5 *insertion element, were moderately increased by 1.7 and 3.0 fold, respectively (Fig. 4). These findings demonstrated that in addition to the presence of an insertion element, two isolates could be differentiated on the basis of expression profiles.

Previous reports indicated that *IS5 *could serve as a mobile transcriptional enhancer in *E. coli *[26]. To determine whether the presence of *IS5 *was responsible for the differences in transcription observed between isolates 'A' and 'B', an *IS5 *gene deletion mutant was constructed using the lambda red recombinase gene replacement system [27]. We set up the following direct pairwise multiple replicated comparisons of transcriptional profiles: 'A' vs. 'B ', 'A' vs. 'AΔ*IS5*', 'AΔ*IS5*' vs. 'B'. In all these comparisons, genes involved in motility and chemotaxis represented the bulk of significant transcriptional differences (more than 90% of all variance in each of the significant lists). Deleting a transposon element from an isolate 'A' reduced the activity of flagella regulon on average by a factor of two. In contrast, the flagella transcripts were still more abundant in Δ*IS5 *strain than in a 'B' isolate, approximately 4 fold on average, suggesting that the presence of an *IS5 *element does not fully explain the difference in transcriptional activity of the motility regulon between two isolates.

A variety of molecular mechanisms are known to regulate the expression of motility and chemotaxis genes in *E. coli *[28]. To investigate other possible molecular causes that might be responsible for the differences in transcriptional activity between isolates 'A' and 'B', we compared sequences of known transcriptional regulators and some of their expected target promoters in two isolates. We have determined that the sequence of *flhD*, *flhC*, *fliA*, *flgM *and *lhrA *are identical between two isolates (data not shown). Detailed PCR analysis of every single motility and chemotaxis operon and intergenic sequences also revealed no discernable differences between two isolates (data not shown).

## Discussion

Herein we presented a reliable and efficient method for linear mapping of mobile elements using whole-genome DNA microarrays. In summary, following DNA microarray hybridization the algorithm to find DNA sequences physically juxtaposed with the sequence of interest is:

1. Calculate the median rank for each gene and sort them in ascending order;

2. Estimate false discovery rates and use them to select a cut-off for significant genes;

3. Filter significant genes by using information about their neighbours

We used sequence data and verification experiments to evaluate the performance of our method. The results were very encouraging. Without any prior knowledge, we could identify positions of all known *IS5 *elements, and could determine a previously unknown insertion site with high sensitivity and specificity.

Another unique property of this approach is that we used single channel cDNA microarray hybridization without a reference channel. Although having reference channel could potentially control the variation of measurement for each spot, which reference sample should be used is sometimes very problematic [29,30]. Using single channel array hybridization could circumvent the need for an often artificial and inadequate reference as well as it can substantially reduce the cost of microarray experiments. Based on our results, single channel microarray experiments can be used to reliably predict the location of insertion sequences on the chromosome, but we need to pay an additional attention to the choice of normalization methods and test statistics. Traditional global normalization and intensity based test statistics may not be applicable here. Based on the presented results, using rank based statistics (such as median of ranks or rank product) can yield the best results. In the future, we will explore more the applicability of single channel microarray experiments as well as the related normalization and analytical issues in different biological contexts.

Compared to the discriminant analysis by [9], a big advantage of the proposed algorithm is that we do not have to have any a priori knowledge about the location of insertion elements. Instead, our algorithm relies only on the microarray data to predict the locations of a sequence of interest. This property allowed us to identify a differentially localized *IS5 *element in one bacterial isolate relative to another, where most of *IS5 *elements had similar (if not identical) locations. The assumption behind the discriminant analysis [9] is that there exists a relatively fixed relationship between the distance from an insertion sequence and the fluorescent intensities of neighbouring probes. This assumption is stronger than ours. Additional advantage of our algorithm is the ease of implementation. Unlike our analysis, the discriminant analysis gives a score for each gene representing the probability that a gene corresponds to a true insertion site. However, we can use estimated FDR to decide the cut-off and use the filtering procedure to screen out possible false positive genes that greatly improves predictive power of the analysis.

DNA microarrays have been successfully used to study relative abundances of RNA and DNA, and in this paper we presented a microarray application for mapping insertion elements in microbial genomes. Here, we discuss how knowledge about the distribution of insertion elements in a bacterial genome leads to important biological insights. Mapping and comparing the distribution of *IS5 *elements in a laboratory strain *E. coli *MG1655 led to the identification of two variants of this strain; these isolates were later found to be phenotypically distinct. Moreover, data obtained from *IS *mapping experiments allowed us to investigate and quantify the effect of *IS5 *on the transcriptional activity of motility and chemotaxis genes in the genome of *E. coli *MG1655.

Initial *IS5 *mapping data predicted two main variants of *E. coli *strain MG1655. These isolates were obtained by analyzing randomly selected colonies from frozen stocks of the strain MG1655 from the ATCC. The only significant difference between these two strains was the presence of an *IS5 *element upstream of the main flagella regulator, *flhDC*. Further biological analyses confirmed the presence of this insertion and showed phenotypic differences between these isolates; one isolate 'A' with an *IS5 *element upstream of the *flhDC *was more motile than isolate 'B', which did not contain such an insertion. Interestingly, when a bacterial population from an original ATCC stock was analyzed for the presence of an *IS5 *in that location we found that about half of the colonies contained an *IS5 *element in vicinity of the *flhDC*. In 100% of the examined cases the presence of the element correlated with higher motility of the cells. Following up on this observation, we investigated the frequency of a spontaneous loss of a motile phenotype. Five thousand colonies descended from a motile variant have been screened and no less motile revertants were found (data not shown). This observation suggests that the heterogeneity observed in the *E. coli *population did not result from an unusually frequent transposition event. Regardless of their origin, such heterogeneities when not accounted for may influence biological interpretation of experimental results where homogeneous offsprings are selected. For instance, before the discovery of the reported heterogeneity in a population of *E. coli *cells, we have observed on multiple occasions that knocking-out random genes results in differential expression of motility/flagella regulon. However, whenever we attempted to complement the knockouts we would discover that differential expression of motility genes was not affected by complementation. In fact, given the 50:50 heterogeneity of a population, there is a 25% chance of encountering motility related differences between a pure parent and a pure offspring and a 50% chance of encountering difference between a homogeneous offspring and an "impure" parent. We also observed instability of expression differences of the motility/chemotaxis regulon when compared biological replicates of liquid cultures grown from separate individual colonies. Given some published reports of motility differences as a major result of gene disruption [31], we would urge caution in interpreting such observations especially in the absence of convincing complementation data.

While this work was in progress, Barker and co-workers reported the finding of motility variants in *E. coli *strain MG1655 [32]. *E. coli *strains with increased motility contained *IS5 *or *IS1 *insertions in the promoter region of *flhDC*. The *IS5 *element present in these strains was located 96 to 99 nucleotides in the upstream direction relative to reported *flhDC *transcription start site. Moreover, the study reported increased expression of the flagella regulators *flhD *and *fliA *as well as of some other genes that are known to be under the control of *flhDC*. These data suggested that *IS5 *was responsible for increasing the level of expression of flagellar genes and producing cells with enhanced motility. Our *IS *mapping and motility assays results were in agreement with these findings. Furthermore, the *IS5 *identified in the strains with increased motility reported in our study were in a somewhat similar position (-311 to -314 bp relative to the *flhDC *transcription start site) and in the same orientation as the *IS5 *found in the strains reported by Barker [32]. Therefore, we decided to investigate whether the presence of *IS5 *upstream of *flhDC *influenced the expression of motility and chemotaxis genes in *E. coli*. Gene expression profiles were obtained for a motile isolate 'A' and its isogenic Δ*IS5 *derivative. The level of expression of flagellar genes in these strains was compared to the less motile isolate 'B'. Our analysis showed that deletion of the *IS5 *element situated upstream of the *flhDC *decreased the level of expression of flagellar genes but did not account for the differences in motility and gene expression observed between the motile and less motile isolates 'A' and 'B'. Moreover, we observed that cells in which an *IS5 *element had been deleted showed increased motility and up-regulation of flagellar genes when compared with less motile cells (isolate 'B'). These results are in agreement with the phenotypes observed in motility assays, strain 'AΔ*IS5*' had a swarming rate that was not very different from the swarming rate of the wild type strain A (Table 4). These findings suggest that *IS5 *can be one of the components of a complex mechanism involved in up-regulation of flagella genes and the increased motility phenotype. If an *IS5 *were solely responsible for the heterogeneous motility observed in *E. coli *strains, then deletion of this transposon would have produced phenotypes similar, within error, to the ones observed in the less motile isolate 'B'. Regulation of the *flhDC *is under control of a variety of mechanisms involving CRP, H-NS, heat shock proteins and transcriptional regulators like LhrA. It is possible that point mutations in any of these regulators or in flagellar regulators not yet identified could explain the observed phenotypic differences. The possibility that genome rearrangements such as deletions or amplifications are responsible for the differential motility phenotype has been ruled out based on comparative genome hybridization and fingerprinting.

**Table 4:** Swarming rates for isolates 'A', 'B', 'A Δ*flhDC*:kan^r^' and 'A Δ*IS5*'. Swarm diameters were measured every hour for 8 hours for overnight colonies inoculated into soft tryptone agar and incubated at 30°C. Average swarm rates and standard errors are shown for at least 15 replicas.

Strain	**Swarm rate (mm h^-1^)**
*E. coli *MG1655 motile (strain A)	4.1 ± 0.1
*E. coli *MG1655 less motile (strain B)	1.3 ± 0.2
*E. coli *MG1655 motile (strain A)(Δ*flhDC*::*kan*^r^)	0.0 ± 0.0
*E. coli *MG1655 motile (strain A) (Δ*IS5*)	3.2 ± 0.1

The results presented in this report provide evidence that supports the role of *IS5 *as a transcriptional enhancer of the *flhDC-*controlled operons. Previously, *IS5 *was linked to the activation and transcriptional regulation of the *bgl *operon in *Escherichia coli *[26]. In this case, transcriptional activation was dependent on the presence of *IS5 *and independent of its position and orientation relative to the promoter region. Our data demonstrated that an *IS5 *element 300 bp upstream of the transcription start site produces a mild enhancement of transcription of flagellar genes that translates into a slight increase in motility.

Interestingly, transcriptional differences between isolates 'A' and 'B' are largely confined to the motility/chemotaxis regulon. If activity of global regulators that are known to modulate transcription of this regulon were perturbed, we would have expected a much wider scope of transcriptional differences between two isolates. If we assume that these narrow regulatory differences are centered on the activity of FlhDC, then we could speculate that a modest 2-fold increase in the *flhDC *transcription results in more than 10-fold up-regulation of the *fliA*. This level of transcriptional amplification translates into phenotypic differences of three to four folds in the swarming rates between the isolates.

The *IS *mapping technique described in this study is a powerful tool that allows gaining insight into biological processes that might otherwise be overlooked when solely relied on available sequence data. This technique could be applied for *de novo *localization of mobile elements or of any other sequence determinant which position needs to be linearly mapped without any prior knowledge. In addition this type of genome-wide mapping combined with DNA cross-linking might provide an efficient way to study three-dimensional chromosomal organization [33].

## Conclusion

Microarray analysis combined with rank statistics is an efficient and reliable method to localized mobile elements in a bacterial genome. Information obtained using this method allowed identification of two variants of *Escherichia coli *strain MG1655 in which the presence of an *IS5 *element produced an enhancement in transcription of flagellar genes and a slight increase in motility. The technique described here can be applied to the linear mapping of any target sequence without prior knowledge of sequence information.

## Methods

### Bacterial strains and growth conditions

*Escherichia coli *strain MG1655 obtained from ATCC was grown on Luria Bertani medium at 37°C in a shaker incubator at 250 rpm. Gene replacement mutants were grown in LB medium containing 30 μg/ml of kanamycin. Strains used for the gene replacement experiments: BW25113/pKD20, BW25141/pKD4 and BT340 were obtained from the *E. coli *Genetic Stock Center (**CGSC) **at Yale University. These strains were grown and maintained in LB medium with the appropriate antibiotics.

### PCR and fluorescent labeling of *IS5 *probes

The probes to map the location of insertion elements in the *E. coli *genome consisted of a 300 bp fragment internal to the *IS5 *gene that was obtained by PCR with the following primers: IS5F-5'-TCGCCAGTTGGTTATCGTTT-3' and IS5R-5'-AGCTGGGTAATCTGCTGCAT-3'. This probe was fluorescently labeled directly by PCR in a reaction containing 500–1000 ng of genomic DNA, 0.5 μM concentration of each primer, 63 μls of sterile distilled water, 10 μls of 10X Thermopol DNA polymerase buffer (New England Biolabs), 5 units of Thermopol DNA Polymerase (New England Biolabs) and dNTPs at the following concentrations 200 μM dATP, 200 μM dGTP, 200 μM dCTP and 100 μM dTTP. Fluorescent labeling of the PCR products was accomplished by adding 100 μM of Cy5 or Cy3 dUTP (Amersham Pharmacia) to each reaction. DNA amplification was done using the following parameters: One cycle of 98°C for 5 minutes; 35 cycles of 98°C for 2 minutes, 55°C for 1 minute and 30 seconds, 72°C for 2 minutes; one last cycle of 72°C for 5 minutes. Reaction products were evaluated by agarose gel electrophoresis and purified using a Millipore Microcon 30 DNA concentration and purification system.

### Microarray experiments

Whole-genome DNA microarrays of *E. coli *were designed, printed and probed as described previously [34]. The microarray consisted of PCR products, within the open reading frames of *Escherichia coli *strain MG1655, robotically spotted on poly-L-lysine-coated glass slides. To ensure the success of PCR amplification and to minimize cross-hybridization we redesigned more than 700 primer pairs from the original set of primers supplied by Sigma-Genosys and their sequences could be downloaded from 

Relative transcript abundances in isolates 'A', 'B' and derivatives have been measured by direct pairwise comparisons in competitive two-color hybridizations. Total RNA samples were extracted from cultures grown on LB medium to OD_600 _of 0.5 using the hot-phenol method [34]. The experimental error of the measurements of RNA abundances was assessed from three independent replicates, where one replicate corresponded to RNA extracted from a culture grown from a separate colony. Following lowess smoothing and variance reduction, differentially expressed genes were identified using two-class comparisons of the adjusted relative expression values by SAM [25] at 1% false discovery rate at the 90^th ^percentile.

Single and two-color hybridizations were carried out in 20–25 ul under a 20 × 20 flat coverslip in hybridization chambers (Monterey Industries) submerged in a 65°C water bath for 5 to 16 hours. Slides were washed sequentially in each of the following solutions: 0.1XSSC and 0.03% SDS, 0.5XSSC, and 0.25XSSC. After the washes, the slides were air dried by centrifugation and scanned with an Axon Genepix 4000B laser scanner at the resolution of 10 um per pixel.

Array CGH (Comparative Genomic Hybridization) was done as a competitive hybridization of fluorescently labeled genomic DNA from isolates 'A' and 'B'. Genomic DNA was extracted using standard procedures [35]. DNA fragments of 700–1000 bps obtained by sonication were used for direct labeling using DNA polymerase I (Klenow fragment). The labeling reaction consisted of 5 μg of DNA, 5 μg of random hexamer (pdN6), 1.5 μl of dNTP mix (0.5 mM dATP, 0.5 mM dCTP, 0.5 mM dGTP and 0.2 mM dTTP), 0.2 mM Cy3 or Cy5 dUTP, 1X Klenow buffer and 8 units of Klenow. Reactions were incubated at 37°C for 2 hours.

### PCR to confirm *IS *element mapping predictions

PCR reactions to confirm the *IS *mapping predictions were done with primers specific for upstream region of *flhDC *and the gene regions of *fliO*/ *fliP *and *fliP/fliQ*. The following primers were used for the PCR reactions: flhDCF3-5'-AGCATGAACGTTTTGTTCCC-3'and yecGR1-5'-CACGCTGCGTAAATCTTCAA-3'; fliPF-5'-ACCTGTCCTTCTCTGGCTGA-3' and fliQR-5'-AACAAGGTGCGGACGTAATC-3'; fliOF-5'-CATTATTGCCCTGATCCTCG-3' and fliPR-5'-TTTAAAGGGCAGAGCAATGG-3'. PCR reactions were done using the following conditions: One cycle at 98°C for 5 minutes; 35 cycles at 98°C for 2 minutes, 60°C for 1 minute and 30 seconds, 72°C for 2 minutes; one last cycle at 72°C for 5 minutes. 500 ng of genomic DNA of isolates 'A' and 'B' was used as a template in these reactions.

### Motility assays

Motility assays were done using tryptone soft agar [36]. This medium consisted of 1% tryptone peptone, 0.5% NaCl and 0.3% Bacto-agar. To assay for motility, either 5 μls of overnight cultures or a fresh *E. coli *colony were gently spotted onto soft agar and incubated at 30°C for 5 hours. Motility was evaluated based on the size of the ring formed by bacteria as they grew on tryptone agar.

### Construction of gene deletion mutants

Deletion mutants for the *flhDC *genes (*flhDC*::kan) were constructed using the lambda red recombinase method described by Datsenko and Wanner [27]. Briefly, *E. coli *K-12 cells containing the pKD20 plasmid were grown in 50 ml SOB medium containing 100 μg/ml of ampicillin and 1 mM arabinose at 30°C. Once the cells reached OD_600 _= 0.6, the culture was concentrated 100 fold by centrifugation and washed once with an equal volume of cold sterile distilled water. Subsequent washes were done twice using half the volume of cold sterile distilled water. The final cell pellets were resuspended in 150 μl of ice cold sterile 10% glycerol. The linear DNA fragments used for gene disruptions were obtained by polymerase chain reaction with plasmid pKD4 as a template and the following primers: flhDCH1P1-5'-TTAAACAGCCTGTACTCTCTGTTCATCCAGCAGTTGTGGGGTGTAGGCTGGAGCTGCTTC-3' and flhDCH2P2C-5'-GTGGGAATAATGCATACCTCCGAGTTGCTGAAACACATTTCATATGAATATCCTCCTTAG-3'. Perkin Elmer high fidelity XL DNA polymerase was used for the amplification of the DNA fragments necessary for gene disruptions. The reaction mixtures and thermo-cycling parameters used have been described by the manufacturer (Perkin Elmer, CA). To do gene disruptions, 50 μl of electrocompetent cells and 3 μl (150 ng) of the linear PCR product were placed in a BioRad gene pulser 0.1 cm gap electroporation cuvette. Introduction of linear DNA into cells was accomplished by electroporation at 1.8 Kv for 5 seconds. Shocked cells were recovered by adding 1 ml of SOC medium and by incubating at 37°C for 90 minutes in a lab line "Cell-Grow" tissue culture rotator. After recovery cells were plated on LB plates containing 30 μg/ml of kanamycin. Candidate mutants were verified by PCR analysis using primers specific for the disrupted gene and the kanamycin cassette present in pKD4. *IS5 *gene deletion mutants were constructed using the procedure described above and the following primers: *IS5*H1P1-5'-CAGATAAGCTATTTTTAAACAGACACTTACCGCACAACAAGTGTAGGCTGGAGCTGCTTC-3' and *IS5*H2P2-5'-AACATTAAGTTGATTGTTGCCTTTCTTTGTATTTAATTAGCATATGAATATCCTCCTTAG-3'.

The kanamycin resistance cassette used to construct the *flhDC *and *IS5 *gene replacements was removed by expressing FLP recombinase from the helper plasmid (pCP20), after its transformation into the gene replacement mutants [27].

### Data Analysis

#### General considerations

Based on the experiment procedure, we can hypothesize the following: (1) the neighbours of *IS5 *have higher intensities than non-neighbours, (2) the closer to *IS5 *a gene is, the higher the intensity of a corresponding element on the array, (3) in the control experiment where no primer extension outside of the element is allowed, only those elements on the array that correspond to the *IS5 *sequence will have high intensities, its neighbours should not have high intensities. We predicted the neighbours of *IS5 *elements scattered across the genome based on these hypotheses, i.e. we identified genes with significant increase in intensity. We used GenBank sequence information and the follow-up verification experiments to evaluate the predicted neighbours and thereby assessed the reliability of whole-genome microarray screening experiments in establishing linear linkages of defined sequence markers. In order to identify the neighbours, we first ranked the genes by a summary ranking statistic from higher intensities to lower intensities; then chose a cut-off value and regarded the genes above the cut-off as significant; finally we filtered some possible false positive genes by using information about neighboring genes.

#### Data pre-processing and ranking statistics

In our analysis we surveyed fluorescent signals from 4281 target sequences representing more than 98% of protein encoding genes of the *E. coli *genome. We used log_2 _transformation and without background correction. For normalization, we tried both global normalization (*x*_*norm *_= *x*-*median*) and scale normalization (*x*_*norm *_= (*x*-*mean*)/*sd*) where *x *is log_2 _(intensity) and *sd *is a standard deviation.

In microarray data analysis, different ranking statistics may give very different lists of significant gene [37], [38]. For traditional two-channel cDNA microarray analysis, SAM *t*-statistic [25] generally performs well in identifying differential expressed genes. We also evaluated the performance of other commonly used ranking statistics such as sample mean and Student *t*-statistic (standardized mean statistic). All of these three statistics use intensities directly. Raychaudhuri [19] showed that in their discriminant analysis using rank of intensities predicted the insertion site better than using intensities directly. Therefore we also tried using other two rank-based statistics: rank-product (the geometric mean of ranks of each replicate for each gene) and median rank (the median of ranks of each replicate). We used the sequence data to compare the quality of predictions that were made on the basis of these different statistics.

#### False discovery rate estimation

We used the false discovery rate (FDR) [39,40] to decide the cut-off values for significant genes. FDR is an alternative to controlling the false positive rate (type I error), and is defined as the expected proportion of false positive genes (FP) among total positive genes (TP); the observed FP/TP ratio is often used to estimate FDR. To calculate FDR, we used a permutation method to estimate the false positive number FP. Under the null hypothesis, we can generate a permuted data set. Specifically, for the rank based statistics, under null hypothesis, the rank of each gene is from a uniform distribution (1,*G*), where *G *is the total number of genes in the experiments. So the permutated data set is just a random sample from number 1 to *G*. For intensities based statistics, the permutation scheme can be found elsewhere [25,38]. We did 100 permutations for each gene. The false positive number from each permutation is the number of genes that counted as significant genes from the permuted data. The average of the false positive numbers over 100 permutations is calculated as FP. The number of genes that counted as significant genes from the original data is regarded as TP, and we estimate FDR = FP/TP [41]. Note that a more elaborate estimator of FDR, namely FDR = π_0_FP/TP with π_0 _as the prior probability of null hypothesis being true, has been proposed [42]. Since π_0 _is a constant and close to 1 in the context of our analysis, we reasoned that using this estimator would not significantly influence our results.

#### Filtering genes using information from the neighbours

Based on our hypothesis, for a true neighbour gene, not only it should have high rank intensity itself, but its neighbour should also have relatively high intensity, i.e. there should exist an association between the intensities of a true significant gene and its neighbours. If the gene is not truly significant, the intensities will be independent between the gene and its neighbour. By chance alone we can falsely identify the genes that are not true significant genes, but the chance of falsely identifying two or more neighbouring genes at the same time is greatly decreased. To formalize this idea, we used hypergeometric distribution to get the *P*-values. Suppose, we have *G *genes, top *m *genes are identified as significant genes, and we define the upstream and downstream 5 genes as the neighbours of gene *i *(so altogether there are 11 neighbour genes including the gene *i *itself). The number of genes from these 11 genes appeared in top *m *gene list will follow a hypergeometric distribution. If we choose *m *= 100, then the *P*-value for identifying one gene among these 11 genes will be 0.23, for identifying two genes will be 0.026 and for three genes will be 0.002. Thus we will deem the genes with at least two neighbours identified together in top *m *list as the significant genes (the neighbours of *IS5*), the genes with one neighbour identified in top *m *list as possible significant genes, and the genes identified alone without any neighbours appeared in top *m *list as possible false significant genes.

## List of Abbreviations

FDR: false discovery rate; TP: total positives; RP: rank product; MR: median rank; IS: insertion elements

## Authors' contributions

BMMV designed and performed all the biological experiments presented in this paper and prepared the manuscript. Yang Xie designed and performed statistical analysis and wrote the data analysis section of this article. WP provided advise and contributed to the statistical part of this work. ABK proposed and supervised the project, carried out parts of the statistical analysis, prepared and edited the final version of the manuscript.

## References

[B1] Mahillon J, Chandler M (1998). Insertion sequences. Microbiol Mol Biol Rev.

[B2] Nagy Z, Chandler M (2004). Regulation of transposition in bacteria. Res Microbiol.

[B3] Bennett PM (2004). Genome plasticity: insertion sequence elements, transposons and integrons, and DNA rearrangement.. Methods Mol Biol.

[B4] Schneider D, Duperchy E, Coursange E, Lenski RE, Blot M (2000). Long-term experimental evolution in Escherichia coli. IX. Characterization of insertion sequence-mediated mutations and rearrangements.. Genetics.

[B5] Kalia A, Mukhopadhyay AK, Dailide G, Ito Y, Azuma T, Wong BC, Berg DE (2004). Evolutionary dynamics of insertion sequences in Helicobacter pylori.. J Bacteriol.

[B6] Moran NA, Plague GR (2004). Genomic changes following host restriction in bacteria.. Curr Opin Genet Dev.

[B7] Zhong S, Khodursky AB, Dykhuizen D, Dean AM (2004). Evolutionary genomics of ecological specialization. Proc Natl Acad Sci USA.

[B8] van Soolingen D, de Haas PE, Hermans PW, Groenen PM, van Embden JD (1993). Comparison of various repetitive DNA elements as genetic markers for strain differentiation and epidemiology of Mycobacterium tuberculosis.. J Clin Microbiol.

[B9] Kivi M, Liu X, Raychaudhuri S, Altman RB, Small PM (2002). Determining the genomic locations of repetitive DNA sequences with a whole-genome microarray: IS6110 in Mycobacterium tuberculosis.. J Clin Microbiol.

[B10] Green L, Miller RD, Dykhuizen DE, Hartl DL (1984). Distribution of DNA insertion element IS5 in natural isolates of Escherichia coli.. Proc Natl Acad Sci USA.

[B11] Ochman H, Gerber AS, Hartl DL (1988). Genetic applications of an inverse polymerase chain reaction.. Genetics.

[B12] Papadopoulos D, Schneider D, Meier-Eiss J, Arber W, Lenski RE, Blot M (1999). Genomic evolution during a 10,000-generation experiment with bacteria. Proc Natl Acad Sci USA.

[B13] Zhong S, Deam AM (2004). Rapid identification and mapping of insertion sequences in Escherichia coli genomes using vectorette PCR. BMC Microbiol.

[B14] DeRisi JL, Iyer VR, Brown PO (1997). Exploring the metabolic and genetic control of gene expression on a genomic scale.. Science.

[B15] Pollack JR, Perou CM, Alizadeh AA, Eisen MB, Pergamenschikov A, Williams CF, Jeffrey SS, Botstein D, Brown PO (1999). Genome-wide analysis of DNA copy-number changes using cDNA microarrays.. Nat Genet.

[B16] Horak CE, Snyder M (2002). ChIP-chip: a genomic approach for identifying transcription factor binding sites.. Methods Enzymol.

[B17] Iyer V, Horak C, Scafe C, Bostein D, Snyder M, Brown PO (2001). Genomic binding sites of the yeast cell-cycle transcription factors SBF and MBF.. Nature.

[B18] Gitan RS, Shi H, Chen CM, Yan PS, Huang TH (2002). Methylation-specific oligonucleotide microarray: a new potential for high-throughput methylation analysis.. Genome Res.

[B19] Raychaudhuri S, Stuart JM, Liu X, Small PM, Altman RB (2000). Pattern recognition of genomic features with microarrays: site typing of Mycobacterium Tuberculosis strains. Proc Int Cont Intell Syst Mol Biol.

[B20] Blattner FR, Plunkett G, Bloch CA, Perna NT, Burland V, Riley M, Collado-Vibes J, Glasner JD, Rode CK, Mayhew GF, Gregor J, Davis NW, Kirkpatrick HA, Goeden MA, Rose DJ, Mau B, Shao Y (1997). The complete genome sequence of Escherichia coli K-12.. Science.

[B21] Soutourina O, Kolb A, Krin E, Laurent-Winter C, Rimsky S, Danchin A, Bertin PN (1999). Multiple control of flagellum biosynthesis in Escherichia coli: role of H-NS protein and the cyclic AMP-catabolite activator protein complex in transcription of the flhDC master operon.. J Bacteriol.

[B22] Schoner B, Kahn M (1981). The nucleotide sequence of IS5 from Escherichia coli.. Gene.

[B23] Dombek PE, Johnson LK, Zimmerley ST, Sadowsky MJ (2000). Use of repetitive DNA sequences and the PCR To differentiate Escherichia coli isolates from human and animal sources.. Appl Environ Microbiol.

[B24] Wolfinger RD, Gibson G, Wolfinger ED, Bennett L, Hamadeh H, Bushel P, Afshari C, Paules RS (2001). Assessing gene significance from cDNA microarray expression data via mixed models.. J Comput Biol.

[B25] Tusher VG, Tibshirani R, Chu R (2001). Significance analysis of microarrays applied to ionizing radiation response. Proc Natl Acad Sci U S A.

[B26] Schnetz K, Rak B (1992). IS5: A mobile enhancer of transcription in Escherichia coli. Proc Natl Acad Sci USA.

[B27] Datsenko KA, Wanner BL (2000). One-step inactivation of chromosomal genes in Escherichia coli K-12 using PCR products.. Proc Natl Acad Sci U S A.

[B28] Soutourina OA, Bertin PN (2003). Regulation cascade of flagellar expression in Gram-negative bacteria.. FEMS Microbiol Rev.

[B29] Talaat AM, Howard ST, Hale W, Lyons R, Garner H, Johnston SA (2002). Genomic DNA standards for gene expression profiling in Mycobacterium tuberculosis.. Nucleic Acids Res.

[B30] Williams BA, Gwirtz RM, Wold BJ (2004). Genomic DNA as a cohybridization standard for mammalian microarray measurements.. Nucleic Acids Res.

[B31] Oshima T, Aiba H, Masuda Y, Kanaya S, Sugiura M, Wanner BL, Mori H, Mizuno T (2002). Transcriptome analysis of all two-component regulatory system mutants of Escherichia coli K-12. Mol Microbiol.

[B32] Barker CS, Prug BM, Matsumura P (2004). Increased motility of Escherichia coli by insertion sequence element integration into the regulatory region of the flhDC operon. J Bacteriol.

[B33] Dekker J, Rippe K, Dekker M, Kleckner N (2002). Capturing chromosome conformation.. Science.

[B34] Khodursky AB, Bernstein JA, Peter BJ, Rhodious V, Wendisch VF, Zimmer DP (2003). Escherichia coli spotted double-strand DNA microarrays:RNA extraction, labeling, hybridization, quality control and data management. Methods Mol Biol.

[B35] Sambrook J, Russell DW (2001). Molecular Cloning: A Laboratory Manual.

[B36] Alder J (1966). Chemotaxis in bacteria. Science.

[B37] Lonnstedt I, Speed T (2002). Replicated microarray data.. Statistica Sinica.

[B38] Xie Y, Jeong KS, Pan W, Khodursky AB, Carlin B (2004). A case study on choosing normalization methods and test statistics for two-channel microarray data. Comp Funct Genom.

[B39] Efron B, Tibshirani R, Storey JD, Tusher VG (2001). Empirical Bayes analysis of a microarray experiment.. Journal of the American Statistical Association.

[B40] Benjamini Y, Hochberg Y, Storey JD, Tibshirani R (1995). Controlling the false discovery rate: a practical and powerful approach to multiple testing. JRSS-B.

[B41] Pan W (2003). On the use of permutation in and the performance of a class of nonparametric methods to detect differential gene expression.. Bioinformatics.

[B42] Storey JDTR (2003). Statistical significance for genome-wide experiments.. Proc Natl Acad Sci.

